# Supplementary material for: Experimental quantum state discrimination using the optimal fixed rate of inconclusive outcomes strategy
Source: Sci Rep. 2022 Oct 15;12:17312. doi: 10.1038/s41598-022-22314-w (PMC9569384; doi:10.1038/s41598-022-22314-w)
Supplement: Supplementary file 1 — Supplementary Information. [file 41598_2022_22314_MOESM1_ESM.pdf]

Supplementary Information: Experimental quantum state discrimination using the optimal fixed rate of inconclusive outcomes strategy

Santiago Gómez[1,2], Esteban S. Gómez[1,2,*], Omar Jiménez[3,+], Aldo Delgado[1,2], Stephen P. Walborn[1,2], and Gustavo Lima[1,2]

[1]Departamento de Física, Universidad de Concepción, casilla 160-C, Concepción, Chile
[2]Millennium Institute for Research in Optics, Universidad de Concepción, casilla 160-C, Concepción, Chile
[3]Centro de Óptica e Información Cuántica, Facultad de Ciencias, Universidad Mayor, Camino La Pirámide N°5750, Huechuraba, Santiago, Chile
[*]estesepulveda@udec.cl
[+]omar.jimenez@umayor.cl

POVM elements for FRIO discrimination

The POVM elements used in the FRIO discrimination have been labeled as Π_1, Π_2 and Π_0, where $\Pi_{1(2)}$ corresponds to the successful (failed) identification of the state $|\phi_{1(2)}\rangle$, and Π_0 is associated to the inconclusive outcome. The general expression for these POVM elements is given by rank-1 unnormalized projectors

$$\Pi_1 = \left(\frac{\sqrt{p_1 + r_2 - 2s\sqrt{p_1 r_2}}}{\sqrt{1-s^2}}\right)^2 |\psi_1\rangle\langle\psi_1| \tag{1}$$

$$\Pi_2 = \left(\frac{\sqrt{r_1 + p_2 - 2s\sqrt{r_1 p_2}}}{\sqrt{1-s^2}}\right)^2 |\psi_2\rangle\langle\psi_2| \tag{2}$$

$$\Pi_0 = \left(\frac{\sqrt{q_1 + q_2 - 2s\sqrt{q_1 q_2}}}{\sqrt{1-s^2}}\right)^2 |\psi_0\rangle\langle\psi_0| \tag{3}$$

where

$$|\psi_1\rangle = \frac{b(\sqrt{p_1}+\sqrt{r_2})}{\sqrt{p_1 + r_2 - 2s\sqrt{p_1 r_2}}}|0\rangle + \frac{a(\sqrt{p_1}-\sqrt{r_2})}{\sqrt{p_1 + r_2 - 2s\sqrt{p_1 r_2}}}|1\rangle,$$
$$|\psi_2\rangle = \frac{b(\sqrt{r_1}+\sqrt{p_2})}{\sqrt{r_1 + p_2 - 2s\sqrt{r_1 p_2}}}|0\rangle + \frac{a(\sqrt{r_1}-\sqrt{p_2})}{\sqrt{r_1 + p_2 - 2s\sqrt{r_1 p_2}}}|1\rangle,$$
$$|\psi_0\rangle = \frac{b(\sqrt{q_1}+\sqrt{q_2})}{\sqrt{q_1 + q_2 - 2s\sqrt{q_1 q_2}}}|0\rangle + \frac{a(\sqrt{q_1}-\sqrt{q_2})}{\sqrt{q_1 + q_2 - 2s\sqrt{q_1 q_2}}}|1\rangle.$$

Here, a and b are the coefficients of the non-orthogonal states to be discriminated $|\phi_{1(2)}\rangle = a|0\rangle \pm b|1\rangle$. $s = a^2 - b^2$ is the inner product between $|\phi_1\rangle$ and $|\phi_2\rangle$, and $p_{1,2}$, $r_{1,2}$, $q_{1,2}$ are the success, error and inconclusive probabilities for the discrimination procedure of $|\phi_{1(2)}\rangle$. Clearly, these POVM elements satisfy the completeness relation $\Pi_1 + \Pi_2 + \Pi_0 = \mathbb{1}$. This POVM can describe any strategy between unambiguous discrimination and minimal error. For instance, when $q_1 = q_2 = 0$, the minimal error discrimination is recovered ($\Pi_0 = 0$ while Π_1 is orthogonal to Π_2).

To implement the POVM elements experimentally, we recall the Neumark dilation theorem, which states that a generalized measurement on a quantum state of dimension d can be described as a projective measurement performed on a larger Hilbert space[1]. Since the non-orthogonal states are encoded in the polarization degree of freedom of single photons, we resort to a two-path Sagnac interferometer to coherently couple the polarization with spatial path modes, increasing the dimensionality of the quantum state. After passing single photons through the interferometer, we can obtain three spatial modes coupled with different polarization modes defined by a specific operation inside the interferometer. This process can be described by a total unitary transformation given by $U = C(\theta_3)\cdot CNOT \cdot C(\theta_2)\cdot CNOT \cdot C(\theta_1)$, where θ_1, θ_2 and θ_3 are the angles of the wave-plates placed at the interferometer[2–4] (see the main text for more details). Then, the three POVM elements can be written

in terms of these wave-plate angles and are given by

$$\Pi_1 = \begin{pmatrix} (\cos\theta_1\cos\theta_2\sin\theta_3 - \cos\theta_3\sin\theta_1)^2 & (\cos\theta_1\cos\theta_2\sin\theta_3 - \cos\theta_3\sin\theta_1)(\cos\theta_1\cos\theta_3 + \cos\theta_2\sin\theta_1\sin\theta_3) \\ (\cos\theta_1\cos\theta_2\sin\theta_3 - \cos\theta_3\sin\theta_1)(\cos\theta_1\cos\theta_3 + \cos\theta_2\sin\theta_1\sin\theta_3) & (\cos\theta_1\cos\theta_3 + \cos\theta_2\sin\theta_1\sin\theta_3)^2 \end{pmatrix},$$

$$\Pi_2 = \begin{pmatrix} (\cos\theta_1\cos\theta_2\cos\theta_3 + \sin\theta_1\sin\theta_3)^2 & (\cos\theta_2\cos\theta_3\sin\theta_1 - \cos\theta_1\sin\theta_3)(\cos\theta_1\cos\theta_2\cos\theta_3 + \sin\theta_1\sin\theta_3) \\ (\cos\theta_2\cos\theta_3\sin\theta_1 - \cos\theta_1\sin\theta_3)(\cos\theta_1\cos\theta_2\cos\theta_3 + \sin\theta_1\sin\theta_3) & (\cos\theta_2\cos\theta]_3\sin\theta_1 - \cos\theta_1\sin\theta_3)^2 \end{pmatrix},$$

$$\Pi_0 = \begin{pmatrix} \cos\theta_1^2\sin\theta_2^2 & \cos\theta_1\sin\theta_1\sin\theta_2^2 \\ \cos\theta_1\sin\theta_1\sin\theta_2^2 & \sin\theta_1^2\sin\theta_2^2 \end{pmatrix}.$$

References

1. Nielsen, M. A. & Chuang, I. L. *Quantum computation and quantum information* (Cambridge University Press, New York, 2010), 10th anniversary edn.
2. Gómez, E. S. *et al.* Device-Independent Certification of a Nonprojective Qubit Measurement. *Phys. Rev. Lett.* **117**, 260401, DOI: 10.1103/PhysRevLett.117.260401 (2016).
3. Gómez, S. *et al.* Experimental nonlocality-based randomness generation with nonprojective measurements. *Phys. Rev. A* **97**, 040102, DOI: 10.1103/PhysRevA.97.040102 (2018).
4. Gómez, S. *et al.* Experimental investigation of partially entangled states for device-independent randomness generation and self-testing protocols. *Phys. Rev. A* **99**, 032108, DOI: 10.1103/PhysRevA.99.032108 (2019).
